# Triglycerides to high-density lipoprotein cholesterol ratio is superior to triglycerides and other lipid ratios as an indicator of increased urinary albumin-to-creatinine ratio in the general population of China: a cross-sectional study

**DOI:** 10.1186/s12944-021-01442-8

**Published:** 2021-02-15

**Authors:** Jing Xue, Yuxia Wang, Bing Li, Songyan Yu, Anping Wang, Weiqing Wang, Zhengnan Gao, Xulei Tang, Li Yan, Qin Wan, Guijun Qin, Lulu Chen, Guang Ning, Yiming Mu

**Affiliations:** 1 Medical School of Chinese PLA, Beijing, China; 2 Department of Endocrinology, The First Medical Center of Chinese PLA General Hospital, No.28 Fuxing Road, Haidian district, Beijing, 100853 China; 3 Shanghai Jiaotong University Affiliated Ruijin Hospital, Shanghai, China; 4 Center Hospital of Dalian, Dalian, Liaoning China; 5 First Hospital of Lanzhou University, Lanzhou, Gansu China; 6 Sun Yat-sen Memorial Hospital, Sun Yat-sen University, Guangzhou, Guangdong China; 7 Affiliated Hospital of Luzhou Medical College, Luzhou, Sichuan China; 8 Zhengzhou University First affiliated Hospital, Zhengzhou, Henan China; 9 Union Hospital, Tongji Medical College, Huazhong University of Science and Technology, Wuhan, Hubei China

**Keywords:** Cross-sectional study, Microalbuminuria, Dyslipidemia, Urinary albumin-creatinine ratio, Triglycerides, High-density lipoprotein cholesterol

## Abstract

**Background:**

Dyslipidemia contributes to the pathogenesis of renal dysfunction. Previous research demonstrated that triglycerides (TG), instead of other individual lipid indexes, has a significant link with elevated urinary albumin-to-creatinine ratio (UACR). However, it is unclear whether lipid ratios are superior indicators of increased UACR compared with TG. This research is to determine whether there are close relationships of lipid ratios with UACR in a general population.

**Methods:**

35,751 participants from seven centers across China were enrolled. UACR equal or higher than 30 mg/g was recognized as increased albuminuria. The associations of TG, low-density lipoprotein cholesterol (LDL-C)/ high-density lipoprotein cholesterol (HDL-C), TG/HDL-C and non-high-density lipoprotein cholesterol (non-HDL-C)/HDL-C with increased UACR were evaluated by linear and logistic regression analyses in females and males separately.

**Results:**

There were 3692 (14.8%) female subjects, and 1307 (12.0%) male subjects characterized as having increased UACR. There were significantly differences in TG/HDL-C and non-HDL-C/HDL-C between the normal UACR group and the increased UACR group, while LDL-C/HDL-C was not. Furthermore, linear regression analysis was implemented and showed that TG and TG/HDL-C were both positively related to UACR even after a variety of potential confounders were adjusted regardless of sexes, while the correlation between non-HDL-C/HDL-C and elevated UACR were only significant in females. Further analyses utilizing logistic regression demonstrated that compared with non-HDL-C/HDL-C and TG, TG/HDL-C showed the strongest association with increased UACR (quartile 1 of TG/HDL-C as a reference; OR [95% CI] of quartile 4: 1.28 [1.13–1.44] in women, 1.24 [1.02–1.50] in men) after fully adjusting for potential confounding factors. Stratified analyses revealed that in males who were overweight and in females who were overweight or over 55 years or had prediabetes or prehypertension, TG/HDL-C had significant associations with abnormal UACR.

**Conclusions:**

Compared with TG and other routine lipid ratios, TG/HDL-C is a superior indicator for increased UACR.

**Supplementary Information:**

The online version contains supplementary material available at 10.1186/s12944-021-01442-8.

## Introduction

Renal dysfunction has posed tremendous economic and medical burdens all across the world [1]. The estimated global prevalence of chronic kidney diseases (CKD) reached 9.1% in 2017, and there were 132.3 million cases of CKD in China [1]. Research has confirmed urinary albumin-to-creatinine ratio (UACR) as one well-established parameter to detect renal insufficiency in early phase. Additionally, emerging evidence has uncovered that an increased UACR has a positive link with cardiovascular diseases no matter in diabetic patients [2, 3] or in the general population [4, 5]. Therefore, identifying indicators of increased UACR is of great importance.

Both experimental [6–8] and clinical studies [9–11] have shown that abnormal lipoprotein metabolism is closely related to the onset and progression of CKD. Notably, emerging epidemiologic studies have shown that in comparison to other individual lipid parameters, TG is a preferable indicator of microalbuminuria. Tien and his colleagues found that triglycerides (TG) was significantly related to micro/macroalbuminuria in the diabetic population [10]. Interestingly, one cross-sectional study conducted by our group enrolling a large Chinese general population also found that elevated levels of TG were independently correlated with abnormal UACR, whereas non-high-density lipoprotein cholesterol (non-HDL-C), low-density lipoprotein cholesterol (LDL-C) and total cholesterol (TC) were not [12].

Recently, accumulating evidence has revealed that compared with traditional lipid profiles, some nontraditional lipid indexes are better indicators to assess cardiovascular risks than individual lipid profiles [13, 14]. As cardiovascular diseases and CKD share common risk factors and a similar pathogenesis of vascular endothelium injury [15], the association between lipid ratios and renal dysfunction is also of interest. A study by Wen et al. in the general population documented that non-HDL-C/HDL-C exhibited higher potency in predicting decreased estimated glomerular filtration rate (eGFR) than individual lipid indexes, and so did TG/HDL-C [16]. Among routine lipids ratios, TG/HDL-C has received increasing attention, as a series of studies have identified TG/HDL-C as a practical indicator of some obesity-related disorders, such as insulin resistance [17, 18], fatty liver [19], diabetes mellitus [20], and cardiovascular diseases [21]. Of note, a cohort study from Japan documented that TG/HDL-C had an inverse correlation with CKD, which was defined by low eGFR and a qualitative assessment of proteinuria.

Although the correlation between lipid ratios and eGFR has been evaluated by several studies, research enrolling a large population from communities to examine the connection between lipid ratios and elevated UACR is rare. A cross-sectional study including 9730 Chinese subjects evaluated the associations of isolated lipid profiles and nontraditional lipid parameters with UACR, and the research hinted that TG/HDL-C could be recognized as a preferable index for the assessment of the risk of increased UACR compared with other routine lipid parameters [22]. However, the results should be interpreted prudently, as liver function and eGFR levels remained unadjusted in multivariable logistic regression, and all the enrolled participants were from a single city in China. Therefore, this research aims at elucidating the associations between lipid ratios and increased UACR in a multicenter large general population and determining whether lipid ratios are superior markers of increased UACR compared with TG.

## Methods

### Study population

**Fig. 1:**
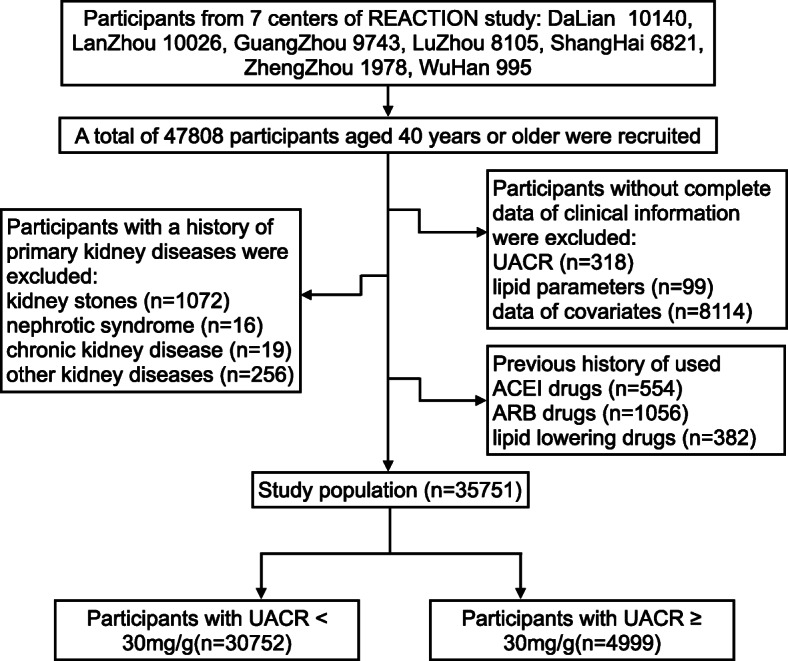
Schematic diagram of study population selection

The data of this study was from the Risk Evaluation of Cancers in Chinese Diabetic Individuals (REACTION study), which was a cohort study involved multiple centers in China and currently ongoing [23]. The study recruited 47,808 subjects from 7 sub-centers in 2012. The sub-centers were composed of Lanzhou, Zhengzhou, Wuhan, Dalian, Luzhou, Guangzhou, and Shanghai. Participants who were diagnosed of primary renal diseases, or with previous usage of lipid lowering drugs, ACEI drugs, or ARB drugs, or with important data missing were excluded (Fig. 1). Finally, 35,751 participants were enrolled in this study.

### Data collection

The subjects completed detailed questionnaires with the assistance of well-trained investigators who had received standardized training before the investigation. The questionnaires documented demographic data, smoking status, alcohol consumption, and medical history.

Blood pressures were measured 3 times with an electronic sphygmomanometer. Pulses were recorded during the blood pressure measurements. Well-trained staff measured and recorded the height and weight of subjects after removal of the overcoat and shoes.

The levels of urine albumin and creatinine were determined by testing first morning spot urine samples.

The participants fasted overnight for more than 10 h before the investigation. The nurses drew the first blood sample at 8–9 am. Then participants received oral glucose tolerance test. Postprandial blood glucose (PBG) levels, aspartate aminotransferase (AST), fasting blood glucose (FBG), serum TG, TC, LDL-C, HDL-C, hemoglobin A1c (HbA1c), alanine aminotransferase (ALT), serum creatinine, gamma-glutamyl transferase (γ-GGT) and fasting insulin were detected by automatic glucose oxidase-peroxidase method.

### Definitions

In the light of the CKD Epidemiology Collaboration equation for “Asian origin” (Supplemental Table 1) [24], eGFR (mL/min/1.73 m^2^) was obtained. Diabetes and prediabetes were diagnosed based on the 2018 American Diabetes Association (ADA) criteria [25].

The definition of body mass index (BMI), lipid ratios, homeostasis model assessment of insulin resistance (HOMA-IR), cardiovascular events, increased UACR, smoking status, drinking status, hypertension and prehypertension were displayed in Supplemental Table 2. Moreover, sex-specific TG quartiles and lipid ratios quartiles were shown in Supplemental Table 3.

### Statistical analysis

The software utilizing for data analyses was SPSS version 23.0 (IBM, Chicago, IL, USA). The quartiles of lipid ratios and TG were calculated in women and men respectively, and subjects were split up into groups based on corresponding quartiles (Supplemental Table 3). Categorical variables were presented as frequencies (percentages). Continuous variables were expressed as means ± standard deviations (SD) or medians (interquartile ranges) according to results of one-sample Kolmogorov-Smirnov test, which was carried out to determine whether variables were normally distributed.

The chi-square test was performed to analysis the differences between categorical variables. The differences in continuous variables between the normal UACR group and the increased UACR group were compared utilizing a two-sample t-test or the Mann-Whitney U test.

The associations between lipid ratios and UACR were explored by Pearson’s correlation analysis. The associations were further evaluated by multivariate linear regression. Furthermore, the correlations of TG and lipid ratios with increased UACR were delineated by logistic regression analyses. All logistic regression analyses were conducted separately by sex. In model 1 plus 2, center, age, ALT, BMI, eGFR, γ-GGT, smoking status, alcohol consumption, and cardiovascular diseases were adjusted. In Model 3, additionally adjustments for HbA1c, systolic blood pressure (SBP), diastolic blood pressure (DBP), pulse, and use of anti-diabetic medication were conducted. HOMA-IR was further adjusted in Model 4. Sensitivity analyses were conducted and the results showed that OR were basically unchanged when education levels and marriage status were added in the logistical regression models 1–4. Moreover, receivers operating characteristic curve (ROC) analyses of TG and lipid ratios for identifying abnormal UACR were conducted. Additionally, the relationships between TG/HDL-C and increased UACR were explored in subgroups stratified by diabetic status, hypertensive status, BMI and age. If two-sided *P* values were ≤ 0.05, the results were considered as statistically significant.

## Results

### Characteristic differences between the normal UACR group and the increased UACR group

In this study, 35,751 participants, including 10,850 men and 24,901 women, were involved. The median UACR was 10.21 (5.99–19.98) mg/g. There were significant differences in individual lipid parameters, lipid ratios and UACR between males and females (all *P* < 0.001) (Supplemental Table 4). Therefore, statistical analyses were conducted in women and men respectively.

As shown in Table 1, female and male participants were categorized by UACR. There were 3692 (14.8%) female participants and 1307 (12.0%) male participants with increased UACR. Individuals with increased UACR showed higher levels of ALT, AST, γ-GGT, blood glucose levels, blood pressure levels, pulse, BMI, HOMA-IR, and Cr and a higher prevalence of CVD events, but lower levels of eGFR. The prevalence of diabetes and hypertension were also notably different between men and women. Furthermore, compared with individuals with normal UACR, those with increased UACR had significantly elevated concentrations of TG, TG/HDL-C and non-HDL-C/HDL-C. Nevertheless, no significant difference in LDL-C/HDL-C between the normal UACR group and the increased UACR group was found.

**Table 1:** Characteristics of the participants categorized by sex and UACR categories

Variables	Total	Female			Male		
		Normal UACR	Increased UACR	***P*** value	Normal UACR	Increased UACR	***P*** value
N	35,751	21,209(85.2%)	3692(14.8%)		9543(88.0%)	1307(12.0%)	
Age (years)	58.3 ± 9.2	56.5 ± 8.7	60.5 ± 10.0	< 0.001	59.2 ± 9.3	62.5 ± 10.1	< 0.001
UACR (mg/g)	10.21(5.99–19.98)	9.29(5.87–15.36)	46.53(35.69–74.70)	< 0.001	7.70(4.83–12.77)	50.52(37.35–89.56)	< 0.001
HDL-C (mmol/L)	1.32 ± 0.33	1.38 ± 0.33	1.31 ± 0.32	< 0.001	1.20 ± 0.30	1.16 ± 0.31	< 0.001
LDL-C (mmol/L)	2.99 ± 0.89	3.06 ± 0.90	2.93 ± 0.91	< 0.001	2.88 ± 0.85	2.77 ± 0.86	< 0.001
TC (mmol/L)	5.08 ± 1.12	5.21 ± 1.12	5.10 ± 1.16	< 0.001	4.84 ± 1.06	4.74 ± 1.09	0.002
TG (mmol/L)	1.37(0.98–1.97)	1.32(0.96–1.89)	1.57(1.11–2.23)	< 0.001	1.38(0.98–2.03)	1.56(1.07–2.33)	< 0.001
Non-HDL-C (mmol/L)	3.76 ± 1.01	3.82 ± 1.02	3.79 ± 1.06	0.066	3.63 ± 0.96	3.58 ± 1.00	0.082
LDL-C/HDL-C	2.34 ± 0.72	2.28 ± 0.69	2.3 ± 0.72	0.147	2.47 ± 0.75	2.47 ± 0.76	0.864
TG/HDL-C	1.07(0.69–1.69)	0.98(0.65–1.53)	1.22(0.81–1.90)	< 0.001	1.19(0.77–1.89)	1.41(0.90–2.26)	< 0.001
Non-HDL-C/HDL-C	2.98 ± 0.97	2.88 ± 0.93	3.02 ± 0.99	< 0.001	3.16 ± 1.03	3.25 ± 1.04	0.003
ALT (U/L)	15.0(11.0–21.0)	14.0(11.0–20.0)	15.0(11.0–21.0)	< 0.001	16.0(12.0–23.0)	17.0(12.0–25.0)	0.002
AST (U/L)	20.0(17.0–25.0)	20.0(17.0–24.0)	21.0(17.0–26.0)	< 0.001	21.0(17.0–25.0)	21.0(17.0–27.0)	0.001
GGT (U/L)	21.0(15.0–32.0)	19.0(14.0–27.0)	20.0(15.0–31.0)	< 0.001	26.0(18.0–39.0)	28.0(19.0–45.0)	< 0.001
FPG (mmol/L)	5.53(5.11–6.18)	5.46(5.08–5.99)	5.67(5.17–6.58)	< 0.001	5.64(5.19–6.37)	6.20(5.40–7.98)	< 0.001
PBG (mmol/L)	7.40(6.04–9.70)	7.19(5.99–9.12)	8.2(6.48–11.33)	< 0.001	7.55(6.00–10.10)	9.33(6.87–13.96)	< 0.001
HbA1c (%)	5.90(5.60–6.30)	5.90(5.60–6.20)	6.00(5.70–6.50)	< 0.001	5.90(5.60–6.30)	6.20(5.80–7.20)	< 0.001
SBP (mmHg)	131.83 ± 20.47	129.56 ± 19.82	138.01 ± 23.00	< 0.001	133.00 ± 19.40	142.72 ± 22.91	< 0.001
DBP (mmHg)	77.36 ± 10.80	75.91 ± 10.28	78.19 ± 11.36	< 0.001	79.56 ± 10.84	82.42 ± 12.42	< 0.001
Pulse (bpm)	79.08 ± 11.48	79.36 ± 11.12	80.09 ± 11.56	< 0.001	77.97 ± 12.04	79.65 ± 12.39	< 0.001
BMI (kg/m^2^)	24.57 ± 3.71	24.44 ± 3.76	24.88 ± 3.99	< 0.001	24.66 ± 3.51	25.20 ± 3.49	< 0.001
HOMA-IR	1.86(1.28–2.73)	1.85(1.29–2.65)	2.15(1.46–3.31)	< 0.001	1.74(1.19–2.61)	2.26(1.42–3.69)	< 0.001
Cr (μmol/L)	65.5(59.4–73.0)	62.3(57.4–67.6)	63.6(58.1–69.6)	< 0.001	75.1(68.3–85.1)	78.4(70.2–90.9)	< 0.001
eGFR (mL/min/1.73^2^)	96.19 ± 14.20	96.97 ± 13.14	91.82 ± 16.44	< 0.001	96.91 ± 14.36	90.53 ± 18.59	< 0.001
Smoking status (%)				0.183			0.949
No	30,712(85.9%)	20,811(98.1%)	3616(97.9%)		5524(57.9%)	761(58.2%)	
Occasional smokers	799(2.2%)	160(0.8%)	23(0.6%)		544(5.7%)	72(5.5%)	
Regular smokers	4240(11.9%)	238(1.1%)	53(1.4%)		3475(36.4%)	474(36.3%)	
Drinking status (%)				< 0.001			< 0.001
No	26,922(75.3%)	18,295(86.3%)	3309(89.6%)		4610(48.3%)	708(54.2%)	
Occasional drinkers	6489(18.2%)	2660(12.5%)	341(9.2%)		3118(32.7%)	370(28.3%)	
Regular drinkers	2340(6.5%)	254(1.2%)	42(1.1%)		1815(19%)	229(17.5%)	
Diabetes (%)	9294(26.0%)	4476(21.1%)	1358(36.8%)	< 0.001	2793(29.3%)	667(51.0%)	< 0.001
Hypertension (%)	11,303(31.6%)	5754(27.1%)	1690(45.8%)	< 0.001	3143(32.9%)	716(54.8%)	< 0.001
Previous CVD (%)	1758(4.9%)	868(4.1%)	264(7.2%)	< 0.001	499(5.2%)	127(9.7%)	< 0.001
Anti-diabetic medication	3145(8.8%)	1366(6.4%)	560(15.2%)	< 0.001	914(9.6%)	305(23.3%)	< 0.001

Continuous data are shown as mean standard deviation or median (interquartile range) and categorical data are shown as frequency (%).

Abbreviations: UACR,urinary albumin creatinine ratio; HDL-C, high-density lipoprotein cholesterol; LDL-C, low-density lipoprotein cholesterol; TC, total cholesterol; TG, triglyceride; non-HDL-C, non-high-density lipoprotein cholesterol; ALT, alanine aminotransferase; AST,aspartate aminotransferase; γ-GGT, gamma-glutamyl transferase; FBG, fasting blood glucose; PBG, postprandial blood glucose; HbA1c, hemoglobin A1c; SBP, systolic blood pressure; DBP, diastolic blood pressure; BMI, body mass index; HOMA-IR, homeostasis model assessment of insulin resistance; Cr, serum creatinine; eGFR, estimated glomerular filtration rate; CVD, cardiovascular disease

### Compared with TG and other lipid ratios, TG/HDL-C displayed a stronger association with increased UACR

Previous research in the Chinese general population found that abnormal level of TG was positively and significantly correlated with increased UACR, while other individual lipid parameters were not [12]. Therefore, our study investigated whether lipid ratios were preferable indicators of increased UACR in the general Chinese population when compared with TG.

First, as shown in Table 2, there was a significant correlation between TG and UACR (Table 2) (*P* < 0.001). TG/HDL-C and non-HDL-C/HDL-C were also significantly linked with UACR when evaluating by Pearson’s correlation analyses. Nonetheless, fully-adjusted linear regression exhibited that non-HDL-C/HDL-C was not significantly linked to UACR in males. TG/HDL-C was inversely related to UACR no matter in males or in females (in females: standardized β = − 0.030, *P* < 0.001; in males: standardized β = 0.022, *P* = 0.025), and the correlation was stronger than TG. Additionally, ROC analyses were performed to determine which lipid parameter was superior in predicting increased UACR (Supplemental Figure 1). Area under the ROC curve (AUC) was computed in ROC analyses. As shown in Supplemental Table 5, the results of ROC analyses conveyed that among TG and lipid ratios, TG/HDL-C obtained the highest AUC in both sexes (AUC of TG/HDL-C, TG, LDL-C/HDL-C, non-HDL-C/HDL-C: 0.595, 0.588, 0.506, 0.538 in women; 0.568, 0.559, 0.502, 0.527 in men), signifying a higher effectiveness of TG/HDL-C in detecting increased UACR in comparison with TG and other lipids ratios.

**Table 2:** Correlation and linear regression analyses of the associations of lipid profiles with UACR

Variables	r	***P*** value	Model 1		Model 2		Model 3	
			Standarized β	***P*** value	Standarized β	***P*** value	Standarized β	***P*** value
**Female**								
TG	0.151	< 0.001	0.086	< 0.001	0.066	< 0.001	0.029	< 0.001
TG/HDL-C	0.162	< 0.001	0.086	< 0.001	0.068	< 0.001	0.030	< 0.001
Non-HDL-C/HDL-C	0.068	< 0.001	0.062	< 0.001	0.045	< 0.001	0.015	0.014
**Male**								
TG	0.094	< 0.001	0.091	< 0.001	0.047	< 0.001	0.019	0.054
TG/HDL-C	0.106	< 0.001	0.089	< 0.001	0.049	< 0.001	0.022	0.025
Non-HDL-C/HDL-C	0.033	0.001	0.058	< 0.001	0.026	0.007	0.007	0.440

Model 1: adjusted for center, age.

Model 2: additionally adjusted for ALT, γ-GGT, BMI, smoking, drinking, cardiovascular disease, eGFR.

Model 3: additionally adjusted for HbA1c, SBP, DBP, pulse, HOMA-IR and use of anti-diabetic medication.

UACR, TG, TG/HDL-C levels were logarithmically transformed to achieve a normal distribution.

Abbreviations: *r* correlation coefficient; *β* regression coefficient; *UACR* urinary albumin-creatinine ratio; *TG* triglyceride; *HDL-C* high-density lipoprotein cholesterol; *non-HDL-C* non-high-density lipoprotein cholesterol; *eGFR* estimated glomerular filtration rate; *ALT* alanine transaminase; *γ-GGT* γ-glutamyl transpeptidase; *BMI* body mass index; *SBP* systolic blood pressure; *DBP* diastolic blood pressure; *HbA1c* hemoglobin A1c; *HOMA-IR* homeostasis model assessment of insulin resistance

To verify the relationships of lipid ratios with UACR, logistic regression analyses were implemented. As shown in Table 3, the unadjusted logistic analyses implied that TG/HDL-C was more closely linked with abnormal UACR than other lipid ratios in both sexes. To further evaluate the association, multiple confounders were adjusted in models 1–3. After adjusting for centers and age in model 1 and subsequently adjusting for ALT, γ-GGT, BMI, eGFR, smoking status, drinking status and CVD events in model 2, the relationship of TG/HDL-C with UACR persisted robust in both sexes. In model 3 and 4, further adjustments for HbA1c, SBP, DBP, pulse, use of anti-diabetic medication and HOMA-IR were performed, and the correlation between TG/HDL-C and increased UACR remained remarkable in females, with an OR (95% CI) of 1.20 (1.07–1.35) for Q2, 1.13 (1.00–1.27) for Q3 and 1.28 (1.13–1.44) for Q4, while the association in males was attenuated but remained significant in Q4, with an OR (95% CI) of 1.24 (1.02–1.50). The relationship between TG and increased UACR was also analyzed. The participants were classified into quartiles according to TG levels. The unadjusted logistic analysis showed that TG was significantly related to abnormal UACR regardless of sexes. However, after adjustments for confounders, the relationship became nonsignificant in males and weakened in females, with an OR (95% CI) of 1.15(1.02–1.29) for Q4. Collectively, compared with TG, TG/HDL-C displayed a stronger correlation with increased UACR.

**Table 3:** Logistic regression models assessing the associations of TG and lipid ratios with increased UACR

	Unadjusted		Model 1		Model 2		Model 3	Model 4		
	OR(95%CI)	***P*** value	OR(95%CI)	***P*** value	OR(95%CI)	***P*** value	OR(95%CI)	***P*** value	OR(95%CI)	***P*** value
**Female**										
TG										
Q1	1.00(reference)		1.00(reference)		1.00(reference)		1.00(reference)		1.00(reference)	
Q2	1.29(1.16–1.44)	< 0.001	1.11(0.99–1.24)	0.083	1.06(0.94–1.19)	0.339	0.99(0.88–1.11)	0.862	0.99(0.88–1.11)	0.819
Q3	1.65(1.48–1.83)	< 0.001	1.26(1.13–1.41)	< 0.001	1.14(1.02–1.28)	0.022	1.03(0.92–1.16)	0.619	1.02(0.91–1.15)	0.688
Q4	2.19(1.98–2.43)	< 0.001	1.64(1.47–1.83)	< 0.001	1.41(1.26–1.57)	< 0.001	1.16(1.03–1.30)	0.013	1.15(1.02–1.29)	0.021
TG/HDL-C										
Q1	1.00(reference)		1.00(reference)		1.00(reference)		1.00(reference)		1.00(reference)	
Q2	1.59(1.42–1.78)	< 0.001	1.33(1.18–1.49)	< 0.001	1.27(1.13–1.43)	< 0.001	1.20(1.07–1.36)	0.002	1.20(1.07–1.35)	0.003
Q3	1.87(1.67–2.08)	< 0.001	1.40(1.24–1.57)	< 0.001	1.27(1.13–1.43)	< 0.001	1.13(1.00–1.27)	0.044	1.13(1.00–1.27)	0.051
Q4	2.55(2.29–2.83)	< 0.001	1.81(1.62–2.03)	< 0.001	1.58(1.41–1.78)	< 0.001	1.29(1.15–1.46)	< 0.001	1.28(1.13–1.44)	< 0.001
Non-HDL-C/HDL-C										
Q1	1.00(reference)		1.00(reference)		1.00(reference)		1.00(reference)		1.00(reference)	
Q2	1.09(0.99–1.21)	0.088	1.08(0.97–1.20)	0.165	1.03(0.92–1.14)	0.644	0.98(0.88–1.10)	0.755	0.98(0.88–1.10)	0.735
Q3	1.17(1.06–1.30)	0.002	1.17(1.06–1.31)	0.003	1.08(0.97–1.21)	0.147	0.98(0.88–1.09)	0.704	0.97(0.87–1.09)	0.642
Q4	1.42(1.28–1.56)	< 0.001	1.51(1.36–1.68)	< 0.001	1.34(1.20–1.49)	< 0.001	1.14(1.02–1.27)	0.024	1.13(1.01–1.26)	0.034
**Male**										
TG										
Q1	1.00(reference)		1.00(reference)		1.00(reference)		1.00(reference)		1.00(reference)	
Q2	1.07(0.90–1.28)	0.454	1.01(0.85–1.21)	0.898	0.90(0.75–1.08)	0.267	0.85(0.70–1.03)	0.093	0.85(0.70–1.03)	0.092
Q3	1.38(1.17–1.64)	< 0.001	1.32(1.11–1.57)	0.001	1.06(0.89–1.27)	0.520	0.97(0.81–1.17)	0.742	0.97(0.80–1.16)	0.704
Q4	1.60(1.36–1.89)	< 0.001	1.66(1.40–1.97)	< 0.001	1.19(0.99–1.43)	0.058	1.06(0.87–1.28)	0.578	1.05(0.87–1.26)	0.643
TG/HDL-C										
Q1	1.00(reference)		1.00(reference)		1.00(reference)		1.00(reference)		1.00(reference)	
Q2	1.20(1.00–1.44)	0.046	1.17(0.97–1.40)	0.100	1.04(0.86–1.25)	0.702	0.98(0.81–1.19)	0.858	0.98(0.81–1.19)	0.831
Q3	1.47(1.23–1.74)	< 0.001	1.40(1.17–1.67)	< 0.001	1.14(0.94–1.37)	0.176	1.05(0.87–1.27)	0.626	1.04(0.86–1.26)	0.671
Q4	1.86(1.58–2.20)	< 0.001	1.90(1.60–2.27)	< 0.001	1.41(1.17–1.70)	< 0.001	1.25(1.03–1.51)	0.024	1.24(1.02–1.50)	0.032
Non-HDL-C/HDL-C										
Q1	1.00(reference)		1.00(reference)		1.00(reference)		1.00(reference)		1.00(reference)	
Q2	1.05(0.89–1.25)	0.546	1.08(0.91–1.29)	0.358	0.97(0.81–1.15)	0.704	0.94(0.79–1.13)	0.528	0.94(0.79–1.13)	0.506
Q3	1.05(0.89–1.24)	0.591	1.12(0.95–1.33)	0.184	0.95(0.79–1.13)	0.552	0.87(0.73–1.05)	0.144	0.87(0.72–1.04)	0.131
Q4	1.29(1.10–1.52)	0.002	1.57(1.32–1.85)	< 0.001	1.21(1.01–1.45)	0.036	1.11(0.92–1.33)	0.287	1.10(0.91–1.32)	0.329

Model 1: adjusted for center, age.

Model 2: additionally adjusted for ALT, γ-GGT, BMI, smoking, drinking, cardiovascular disease, eGFR.

Model 3: additionally adjusted for HbA1c, SBP, DBP, pulse and use of anti-diabetic medication.

Model 4: additionally adjusted for HOMA-IR.

Abbreviations: *UACR* urinary albumin-creatinine ratio; *OR* odds ratio; *CI* confidence interval; *TG* triglyceride; *HDL-C* high-density lipoprotein cholesterol; *non-HDL-C* non-high-density lipoprotein cholesterol; *eGFR* estimated glomerular filtration rate; *ALT* alanine transaminase; *γ-GGT* γ-glutamyl transpeptidase; *BMI* body mass index; *SBP* systolic blood pressure; *DBP* diastolic blood pressure; *HbA1c* hemoglobin A1c; *HOMA-IR* homeostasis model assessment of insulin resistance

### Stratified analyses in subgroups categorized by blood glucose, blood pressure, BMI and age

Stratified multivariable logistic regression analyses were applied in males and females separately. As shown in Table 4, the analyses of female subjects identified an independent link between TG/HDL-C and rising level of UACR in the prehypertension and hypertension strata, both the normal BMI strata and overweight strata, both the age > 55 and age ≤ 55 strata, and the prediabetes strata. However, the association only remained significant in male subjects who were overweight. It is noteworthy that the male subjects in the overweight strata were especially susceptible to increased UACR when they were in the fourth quartile (OR [95%CI: 1.58[1.15–2.17]).

**Table 4:** The logistic regression analyses evaluating the correlation between TG/HDL-C and increased UACR in subgroups

Variable	Q1	Q2	***P*** value	Q3	***P*** value	Q4	***P*** value
		OR (95% CI)		OR (95% CI)		OR (95% CI)	
**Female**							
Blood glucose^a^							
Normal	1.00(reference)	0.99(0.77–1.27)	0.931	1.12(0.86–1.45)	0.396	1.13(0.85–1.51)	0.404
Prediabetes	1.00(reference)	1.34(1.13–1.58)	0.001	1.18(0.99–1.39)	0.060	1.31(1.10–1.56)	0.003
Diabetes	1.00(reference)	1.06(0.83–1.37)	0.632	1.03(0.81–1.31)	0.820	1.22(0.97–1.54)	0.092
Blood pressure^b^							
Normal	1.00(reference)	1.01(0.81–1.26)	0.915	0.97(0.77–1.22)	0.774	0.92(0.71–1.18)	0.496
Prehypertension	1.00(reference)	1.37(1.12–1.68)	0.002	1.22(0.99–1.49)	0.057	1.48(1.21–1.81)	< 0.001
Hypertension	1.00(reference)	1.13(0.92–1.39)	0.234	1.12(0.92–1.36)	0.270	1.32(1.09–1.60)	0.005
BMI^c^							
BMI < 18.5	1.00(reference)	0.92(0.50–1.70)	0.797	0.58(0.24–1.43)	0.237		
18.5 ≤ BMI < 24	1.00(reference)	1.23(1.05–1.45)	0.012	1.09(0.92–1.30)	0.309	1.25(1.05–1.49)	0.014
24 ≤ BMI < 28	1.00(reference)	1.19(0.95–1.47)	0.128	1.16(0.94–1.43)	0.179	1.37(1.11–1.68)	0.003
BMI ≥ 28	1.00(reference)	1.18(0.80–1.74)	0.395	1.31(0.92–1.89)	0.139	1.37(0.96–1.97)	0.083
Age^d^							
≤ 55	1.00(reference)	1.06(0.87–1.28)	0.575	1.10(0.91–1.34)	0.339	1.26(1.03–1.55)	0.023
> 55	1.00(reference)	1.32(1.14–1.55)	< 0.001	1.17(1.00–1.36)	0.050	1.28(1.10–1.49)	0.001
**Male**							
Blood glucose^a^							
Normal	1.00(reference)	1.12(0.68–1.84)	0.655	0.85(0.49–1.48)	0.573	0.98(0.55–1.78)	0.956
Prediabetes	1.00(reference)	0.94(0.70–1.24)	0.643	0.98(0.73–1.32)	0.906	1.12(0.83–1.52)	0.456
Diabetes	1.00(reference)	0.96(0.71–1.30)	0.787	1.17(0.87–1.58)	0.300	1.36(1.01–1.83)	0.041
Blood pressure^b^							
Normal	1.00(reference)	1.05(0.66–1.67)	0.855	1.14(0.71–1.85)	0.585	1.36(0.82–2.26)	0.240
Prehypertension	1.00(reference)	0.98(0.71–1.37)	0.923	0.97(0.70–1.35)	0.867	1.10(0.79–1.54)	0.574
Hypertension	1.00(reference)	0.93(0.71–1.22)	0.600	0.98(0.74–1.29)	0.871	1.18(0.90–1.54)	0.241
BMI^c^							
BMI < 18.5	1.00(reference)	1.14(0.35–3.70)	0.823	0.28(0.03–3.06)	0.298		
18.5 ≤ BMI < 24	1.00(reference)	0.93(0.71–1.23)	0.621	1.01(0.76–1.36)	0.930	0.92(0.66–1.27)	0.598
24 ≤ BMI < 28	1.00(reference)	1.13(0.81–1.58)	0.476	1.24(0.90–1.71)	0.198	1.58(1.15–2.17)	0.005
BMI ≥ 28	1.00(reference)	0.86(0.47–1.55)	0.610	0.96(0.55–1.69)	0.898	1.32(0.76–2.28)	0.322
Age^d^							
≤ 55	1.00(reference)	1.20(0.77–1.88)	0.422	1.04(0.67–1.62)	0.852	1.19(0.78–1.84)	0.419
> 55	1.00(reference)	0.91(0.74–1.13)	0.410	1.03(0.83–1.28)	0.762	1.20(0.97–1.49)	0.101

Model a: center, age, ALT, γ-GGT, BMI, eGFR, cardiovascular disease, smoking, drinking, SBP, DBP, pulse, use of anti-diabetic medication, HOMA-IR.

Model b: center, age, ALT, γ-GGT, BMI, eGFR, cardiovascular disease, smoking, drinking, pulse, HbA1c, use of anti-diabetic medication, HOMA-IR.

Model c: center, age, ALT, γ-GGT, eGFR, cardiovascular disease, smoking, drinking, SBP, DBP, pulse, HbA1c, use of anti-diabetic medication, HOMA-IR.

Model d: center, ALT, γ-GGT, BMI, eGFR, cardiovascular disease, smoking, drinking, SBP, DBP, pulse, HbA1c, use of anti-diabetic medication, HOMA-IR.

Abbreviations: *TG* triglyceride; HDL-C, high-density lipoprotein cholesterol; *UACR* urinary albumin-creatinine ratio; *OR* odds ratio; *CI* confidence interval; *eGFR* estimated glomerular filtration rate; *ALT* alanine transaminase; *γ-GGT* γ-glutamyl transpeptidase; *BMI* body mass index; *SBP* systolic blood pressure; *DBP* diastolic blood pressure; *HbA1c* hemoglobin A1c; *HOMA-IR* homeostasis model assessment of insulin resistance

**Fig. 2:**
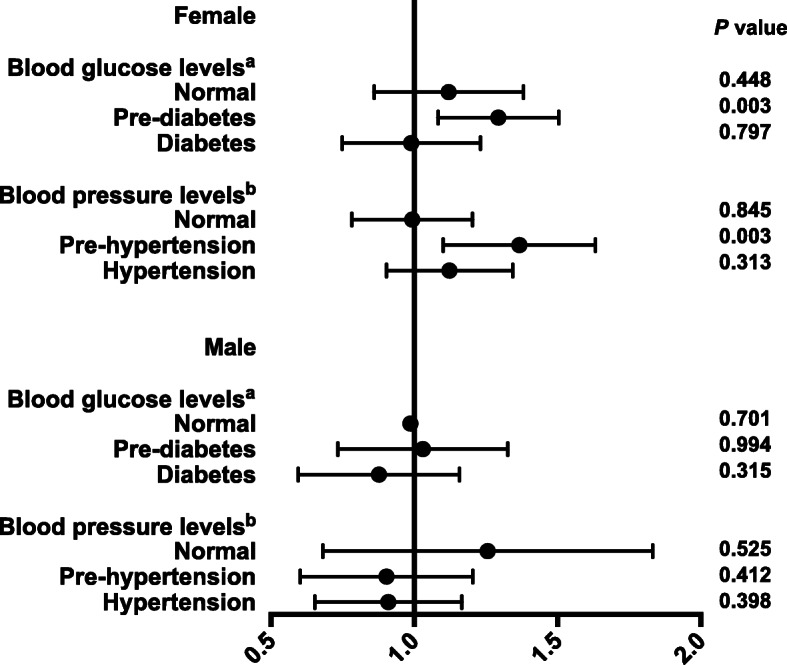
Associations between TG/HDL and increased UACR in participants with normal TG and HDL-C levels

Furthermore, the correlation was assessed in individuals whose TG and HDL-C were both within normal range. The 25th percentile of TG/HDL-C was selected as the cut-off point (0.66 for women, 0.78 for men). Females were divided into two strata according to the cut-off point, and males were also divided into two groups based on corresponding cut-off point. The logistic analyses revealed that even if TG and HDL-C were within the normal range, if TG/HDL-C was equal to or more than 0.66, the risks of increased UACR were significantly increased in female individuals with prediabetes/prehypertension (Fig. 2). However, the association disappeared in males. These results suggested that for women with prediabetes/prehypertension, it was highly necessary to keep serum concentrations of TG and HDL-C under stricter surveillance, thus maintaining a lower TG/HDL-C level.

Participants with TG < 1.7 mmol/L and HDL-C ≥ 1.0 mmol/L were stratified by blood glucose and blood pressure levels. The associations were evaluated by logistic regression analyses. Model a: adjusted for center, age, ALT, γ-GGT, BMI, eGFR, cardiovascular disease, smoking, drinking, SBP, DBP, pulse, use of anti-diabetic medication and HOMA-IR. Model b: adjusted for center, age, ALT, γ-GGT, BMI, eGFR, cardiovascular disease, smoking, drinking, pulse, HbA1c, use of anti-diabetic medication and HOMA-IR.

## Discussion

This study showed that among nontraditional lipid indexes, TG/HDL-C was most closely correlated with abnormal UACR even after fully adjustments for potential confounders in a large general population of Chinese individuals. Furthermore, compared with TG, TG/HDL-C was a preferable indicator to renal dysfunction regardless of sexes. Stratified analyses showed that in female subjects, the correlation of TG/HDL-C with UACR persisted robust in both prehypertension and hypertension strata, both the normal weight and overweight strata, both the age > 55 and age ≤ 55 strata, and the prediabetes strata, while in male subjects, the association remained significant only in the overweight strata. Interestingly, in female individuals with prediabetes/prehypertension, even if TG and HDL were within the normal range, if TG/HDL was equal to or greater than 0.66, the risks of increased UACR were significantly increased. To the best of our knowledge, this is the first multicenter, large sample study about the associations of nontraditional lipid parameters with increased UACR in China.

Dyslipidemia has been regarded as a contributor to the onset and exacerbation of renal insufficiency [6, 8]. The association between lipid ratios and renal insufficiency has attracted researchers’ interest in recent years, as some research demonstrated that compared with individual lipid parameters, some lipid ratios may be better markers for the assessment of cardiovascular diseases [13, 14, 26] that share a similar pathogenesis of vascular endothelium injury and have common risk factors with renal insufficiency [15]. However, studies on the associations of lipid ratios with UACR are limited. A study from Korea manifested that the correlation of TG/HDL-C with abnormal UACR in subjects with normal blood pressure levels existed, but the association disappeared in hypertensive individuals [27]. But the study enrolled only 9094 rural Korean, and some important confounding factors included liver function and eGFR were unadjusted in the logistic regression, which may decrease the validity of the results. Furthermore, a study from a sub-center of the REACTION study found that TG/HDL-C showed a more powerful association with elevated UACR compared with other lipid parameters [22]. However, the study did not carry out fully adjustments for confounders, including HOMA-IR, which was found to be closely related to both UACR [28] and TG/HDL-C [17, 18]. It is noteworthy that compared with the abovementioned studies, this study involved a larger general population from 7 regions of China and adjusted for extensive confounders, which ensured a preferable representation of the general population in China. And unlike abovementioned research, in this study, stratified analyses were conducted in prediabetes and prehypertension subgroups, thus providing a more comprehensive assessment. Our research implied that TG/HDL-C was a more practical marker in evaluating the risk of abnormal UACR in China and, for the first time, identified TG/HDL-C as one useful indicator of abnormal UACR particularly in those with prediabetes or prehypertension.

Various studies have reported the positive relationship of TG with UACR [9, 10, 29]. Our previous study including 34,569 Chinese revealed that elevated TG, instead of other individual lipid parameters, was a practical indictor of increased UACR [12]. Moreover, clinical studies demonstrated that HDL-C concentrations were inversely related to the levels of UACR [9, 30], and experimental observations revealed that HDL-C abnormalities induced endothelial dysfunction, augmented oxidative stress and boosted atherosclerosis, all of which contributed to renal dysfunction [31]. As the influences of both TG and HDL-C on kidney diseases were integrated by TG/HDL-C, it seemed reasonable that TG/HDL-C was a preferable indicator of increased UACR than TG alone, as shown in this research.

Another possible mechanism underlying the link between TG/HDL-C and UACR was the malfunction of insulin signaling pathway. Compelling evidence exhibited that TG/HDL-C was remarkably linked with insulin resistance in various ethnic backgrounds [17, 18]. Insulin signaling pathway is indispensable for podocytes and tubular cells to perform physiological functions normally and efficiently. For example, Welsh et al. [32] found podocytes could dynamically change the structure of the actin cytoskeleton in response to insulin, which played crucial role in keeping glomerular filtration barrier function and avoiding the abnormal urine albumin secretion. Both animal experiments and clinical data have manifested that insulin resistance exerted a negative impact on renal function, including disturbing glomerular haemodynamics and attenuating podocyte funtion, leading to the decreased eGFR and microalbuminuria [32–34]. Therefore it was reasonable to speculate that the malfunction of insulin signaling pathway contributed to the robust association between TG/HDL-C and increased UACR. However, after an adjustment of HOMA-IR in the logistic regression analyses, the associations remained robust in both sexes, which suggested that in addition to insulin resistance, there were other mechanisms contributing to the association.

A possible explanation was the close link between TG/HDL-C and small, dense LDL-C, which was highly atherogenic. Small, dense LDL-C could readily convert to oxidized LDL-C, which induces mitochondrial dysfunction and elevates reactive oxygen species production in podocytes, leading to podocyte injury and a fragmented protein diaphragm architecture [35, 36]. Research identified a negative association between the size of TG/HDL-C and LDL. Consequently, TG/HDL-C was considered to be a reliable index to evaluate the value of small, dense LDL-C in Asian population [37, 38]. Hence, the relationship between TG/HDL-C and microalbuminuria may be attributable to small, dense LDL-C.

The large population of this study allowed an extensive adjustment for confounders and further analyses in subjects with different characteristics. In particular, this study for the first time described the significant link between TG/HDL-C and UACR in a prediabetes and prehypertension population. Interestingly, the association weakened in diabetic or hypertensive participants. It was likely that the strong effect of diabetes and hypertension on renal function [39, 40] covered the link between TG/HDL-C and renal insufficiency. Of note, in females suffered from prediabetes or prehypertension, even if TG and HDL-C levels were both normal, increasing levels of TG/HDL-C were clearly in correlation with the abnormal level of UACR. These results hinted that higher TG/HDL-C probably represented the early phase of abnormal lipid metabolism and subsequent renal injury. As the initiation and progression of CKD is usually insidious and often neglected, this finding highlights the necessity for the population at the borderline of diabetes or hypertension to more strictly control TG and HDL-C levels. More specifically, for female individuals with prediabetes or prehypertension, maintaining TG/HDL-C levels less than 0.66 is probably beneficial to prevent potential renal dysfunction.

### Study strengths and limitations

The study recruited a large general population from different regions of China, and conducted the comprehensive adjustments of covariates and stratified analyses, which were the strengths of this study.

However, this study had several limitations. First, owing to the intrinsic feature of the cross-sectional study, this study can only evaluate the association rather than causality. In addition, research has shown that there are complex interactions between dyslipidemia and renal dysfunction [31, 41], thus further large cohort studies are indispensible to elucidate whether there is a cause-effect association between TG/HDL-C and increased UACR. Second, serum urinary albumin concentration was tested merely in one urine samples. Multiple or 24-h urine samples would definitely provide more accurate and stable data on urine albumin excretion. Nevertheless, the measurement of spot urine samples is widely used in epidemiological surveys owing to its convenience and close correlation with the results of multiple and 24-h urine samples [42, 43]. Third, the interpretation of this study in a younger Chinese population or other ethnic groups should be taken with caution because this study enrolled middle-aged Chinese population. Finally, this study lacked data on apolipoprotein A, apolipoprotein B, remnant-like particle cholesterol and other atherogenic lipid indexes, which could provide more evidence on the link between lipid parameters and renal dysfunction.

## Conclusions

In conclusion, in the Chinese general population, compared with TG and other lipid ratios, TG/HDL-C shows a stronger association with increased UACR, especially in overweight individuals or individuals with prediabetes or prehypertension. These data imply that TG/HDL-C is a useful indicator of renal insufficiency. Clinical medical workers should attach more emphasis on maintaining normal TG/HDL-C levels. More intensive surveillance and stricter control of TG/HDL-C levels are conducive to the prevention of renal dysfunction, especially for populations who are overweight or at the borderline of diabetes or hypertension. In the future, prospective cohort studies in a large population are needed to better elucidate the relationship between TG/HDL-C and increased UACR.

## Supplementary Information

**Additional file 1 Table S1.** The equation for the estimated glomerular filtration rate. **Table S2.** Definition of variables involved in statistical analyses. **Table S3.** Sex-specific TG quartiles and lipid ratios quartiles in all participants. **Table S4.** Characteristics of participants categorized by sex. **Table S5.** Receiver operating characteristic analyses of TG and lipid ratios for identifying increased UACR in men and women.

**Additional file 2 Figure S1.** Receivers operating characteristic (ROC) curves of TG and lipid ratios for identifying increased UACR.

## Data Availability

The datasets analyzed during this study are not freely available for the sake of the participants’ privacy protection but are available from the corresponding author on reasonable request.

## Abbreviations

TG: Triglycerides
UACR: Urinary albumin-creatinine ratio
CKD: Chronic kidney disease
HDL-C: High-density lipoprotein cholesterol
LDL-C: Low-density lipoprotein cholesterol
non-HDL-C: Non-high-density lipoprotein cholesterol
TC: Total cholesterol
OR: Odds ratio
CI: Confidence interval
eGFR: estimated glomerular filtration rate
PBG: Postprandial blood glucose
FBG: Fasting blood glucose
HbA1c: Hemoglobin A1c
ALT: Alanine aminotransferase
AST: Aspartate aminotransferase
γ-GGT: γ-glutamyl transpeptidase
BMI: Body mass index
HOMA-IR: Homeostasis model assessment of insulin resistance
SBP: Systolic blood pressure
DBP: Diastolic blood pressure
SD: Standard deviations
CVD: Cardiovascular disease
ROC: Receiver operating characteristic curve
AUC: Area under the receiver operating characteristic curve
