# Supplementary material for: Triglycerides to high-density lipoprotein cholesterol ratio is superior to triglycerides and other lipid ratios as an indicator of increased urinary albumin-to-creatinine ratio in the general population of China: a cross-sectional study
Source: Lipids Health Dis. 2021 Feb 15;20:13. doi: 10.1186/s12944-021-01442-8 (PMC7883433; doi:10.1186/s12944-021-01442-8)
Supplement: Supplementary file 1 — Additional file 1 Table S1. The equation for the estimated glomerular filtration rate. Table S2. Definition of variables involved in statistical analyses. Table S3. Sex-specific TG quartiles and lipid ratios quartiles in all participants. Table S4. Characteristics of participants categorized by sex. Table S5. Receiver operating characteristic analyses of TG and lipid ratios for identifying increased UACR in men and women. [file 12944_2021_1442_MOESM1_ESM.docx]

Supplemental Table 1. The equation for the estimated glomerular filtration rate.

| **Sex** | **Serum creatinine (μmol/L)** | **Formula (mL/min/1.73 m^2^)** |
| --- | --- | --- |
| Female | ≤61.88 | 151×(Scr/61.88)^−0.328^×(0.993)^age^ |
|  | >61.88 | 151×(Scr/61.88)^−1.210^×(0.993)^age^ |
| Male | ≤79.56 | 149×(Scr/79.56)^−0.415^×(0.993)^age^ |
|  | >79.56 | 149×(Scr/79.56)^−1.210^×(0.993)^age^ |
| Abbreviations: Scr, serum creatinine. The unit of age is years. | | |

Supplemental Table 2. Definition of variables involved in statistical analyses.

| **Variables** | **Definition** |
| --- | --- |
| BMI (Kg/m^2^) | Body weight (Kg) / body height (m) ^2^ |
| HOMA-IR | Fasting plasma glucose (mmol/L) × fasting insulin (mU/L) / 22.5. |
| Non-HDL-C (mmol/L) | TC (mmol/L) − HDL-C (mmol/L) |
| TG/HDL-C | TG (mmol/L) / HDL-C (mmol/L) |
| Non-HDL-C/HDL-C | Non-HDL-C (mmol/L) / HDL-C (mmol/L) |
| LDL-C/HDL-C | LDL-C (mmol/L) / HDL-C (mmol/L) |
| Cardiovascular events | A previous history of stroke, myocardial infarction or coronary heart disease |
| Increased UACR | UACR ≥30 mg/g |
| Smoking status |  |
| Regular smokers | Smoking one or more cigarette/day |
| Occasional smokers | Smoking less than one cigarette/day or less than 7 cigarettes/week |
| Nonsmokers | Currently not smoking |
| Drinking status |  |
| Regular drinkers | Drinking nearly or more once a week. |
| Occasional drinkers | Drinking less than once a week. |
| Nondrinkers | Currently not drinking |
| Hypertension | A medical history of hypertension or SBP ≥ 140 mmHg or DBP ≥ 90 mmHg |
| Pre-hypertension | 120 mmHg ≤ SBP < 140 mmHg or 80 mmHg ≤ DBP< 90 mmHg. |

Abbreviations: BMI, body mass index; HOMA-IR, homeostasis model assessment of insulin resistance; non-HDL-C, non-high-density lipoprotein cholesterol; HDL-C, high-density lipoprotein cholesterol; TG, triglyceride; LDL-C, low-density lipoprotein cholesterol; UACR, urinary albumin creatinine ratio; SBP, systolic blood pressure; DBP, diastolic blood pressure.

Supplemental Table 3. Sex-specific TG quartiles and lipid ratios quartiles in all participants.

| **Variables** | **Q1** | **Q2** | **Q3** | **Q4** |
| --- | --- | --- | --- | --- |
| **Female** |  |  |  |  |
| TG | <0.98 | 0.99-1.35 | 1.36-1.93 | ≥1.94 |
| TG/HDL-C | <0.66 | 0.66-1.00 | 1.01-1.58 | ≥1.59 |
| Non-HDL-C/HDL-C | <2.23 | 2.23-2.80 | 2.81-3.45 | ≥3.46 |
| **Male** |  |  |  |  |
| TG | <0.99 | 1.00-1.39 | 1.40-2.05 | ≥2.06 |
| TG/HDL-C | <0.78 | 0.78-1.21 | 1.22-1.93 | ≥1.94 |
| Non-HDL-C/HDL-C | <2.45 | 2.45-3.07 | 3.08-3.75 | ≥3.76 |

Abbreviations: TG, triglyceride; HDL-C, high-density lipoprotein cholesterol; non-HDL-C, non-high-density lipoprotein cholesterol.

Supplemental Table 4. Characteristics of participants categorized by sex.

| **Variables** | **Total** | **Female** | **Male** | ***P value*** |
| --- | --- | --- | --- | --- |
| N | 35751 | 24901(69.7%) | 10850(30.3%) |  |
| Age (years) | 58.33±9.19 | 57.06±8.99 | 59.57±9.43 | <0.001 |
| UACR (mg/g) | 10.21(5.99-19.98) | 10.94(6.39-21.13) | 8.75(5.19-16.97) | <0.001 |
| HDL (mmol/L) | 1.32±0.33 | 1.37±0.33 | 1.20±0.31 | <0.001 |
| LDL (mmol/L) | 2.99±0.89 | 3.04±0.90 | 2.87±0.86 | <0.001 |
| TC (mmol/L) | 5.08±1.12 | 5.19±1.13 | 4.83±1.06 | <0.001 |
| TG (mmol/L) | 1.37(0.98-1.97) | 1.36(0.98-1.94) | 1.40(0.99-2.06) | <0.001 |
| Non-HDL-C (mmol/L) | 3.76±1.01 | 3.82±1.02 | 3.63±0.97 | <0.001 |
| LDL-C/HDL-C | 2.34±0.72 | 2.28±0.69 | 2.47±0.75 | <0.001 |
| TG/HDL-C | 1.07(0.69-1.69) | 1.01(0.66-1.59) | 1.22(0.78-1.94) | <0.001 |
| Non-HDL-C/HDL-C | 2.98±0.97 | 2.90±0.94 | 3.17±1.03 | <0.001 |
| ALT (U/L) | 15.0(11.0-21.0) | 14.0(11.0-20.0) | 16.0(12.0-23.0) | <0.001 |
| AST (U/L) | 20.0(17.0-25.0) | 20.0(17.0-24.0) | 21.0(17.0-25.0) | <0.001 |
| GGT (U/L) | 21.0(15.0-32.0) | 19.0(14.0-28.0) | 26.0(18.0-40.0) | <0.001 |
| FPG (mmol/L) | 5.53(5.11-6.18) | 5.49(5.09-6.05) | 5.69(5.20-6.50) | <0.001 |
| PBG (mmol/L) | 7.40(6.04-9.70) | 7.30(6.02-9.40) | 7.70(6.08-10.49) | <0.001 |
| HbA1c (%) | 5.90(5.60-6.30) | 5.90(5.60-6.20) | 5.90(5.60-6.30) | <0.001 |
| SBP (mmHg) | 131.83±20.47 | 130.81±20.54 | 134.17±20.10 | <0.001 |
| DBP (mmHg) | 77.36±10.80 | 76.25±10.48 | 79.90±11.09 | <0.001 |
| Pulse (bpm) | 79.08±11.48 | 79.47±11.19 | 78.18±12.09 | <0.001 |
| BMI (kg/m^2^) | 24.57±3.71 | 24.51±3.79 | 24.73±3.51 | <0.001 |
| HOMA-IR | 1.86(1.28-2.73) | 1.88(1.32-2.73) | 1.79(1.21-2.71) | <0.001 |
| Cr (μmol/L) | 65.5(59.4-73.0) | 62.5(57.5-67.8) | 75.4(68.5-85.7) | <0.001 |
| eGFR (mL/min/1.73^2^) | 96.19±14.20 | 96.21±13.80 | 96.14±15.07 | 0.689 |
| Smoking status (%) |  |  |  | <0.001 |
| No | 30712(85.9%) | 24427(98.1%) | 6285(57.9%) |  |
| Occasional smokers | 799(2.2%) | 183(0.7%) | 616(5.7%) |  |
| Regular smokers | 4240(11.9%) | 291(1.2%) | 3949(36.4%) |  |
| Drinking status (%) |  |  |  | <0.001 |
| No | 26922(75.3%) | 21604(86.8%) | 5318(49.0%) |  |
| Occasional drinkers | 6489(18.2%) | 3001(12.1%) | 3488(32.1%) |  |
| Regular drinkers | 2340(6.5%) | 296(1.2%) | 2044(18.8%) |  |
| Previous CVD (%) | 1758(4.9%) | 1132(4.5%) | 626(5.8%) | <0.001 |
| Diabetes (%) | 9294(26.0%) | 5834(23.4%) | 3460(31.9%) | <0.001 |
| Hypertension (%) | 11303(31.6%) | 3859(35.6%) | 7444(29.9%) | <0.001 |
| Anti-diabetic medication (%) | 3145(8.8%) | 1926(7.7%) | 1219(11.2%) | <0.001 |

Continuous data are shown as the mean standard deviation or median (interquartile range), and categorical data are shown as the frequency (%).

Abbreviations: UACR, urinary albumin creatinine ratio; HDL-C, high-density lipoprotein cholesterol; LDL-C, low-density lipoprotein cholesterol; TC, total cholesterol; TG, triglyceride; non-HDL-C, non-high-density lipoprotein cholesterol; ALT, alanine aminotransferase; AST, aspartate aminotransferase; γ-GGT, gamma-glutamyl transferase; FBG, fasting blood glucose; PBG, postprandial blood glucose; HbA1c: hemoglobin A1c; SBP, systolic blood pressure; DBP, diastolic blood pressure; BMI, body mass index; HOMA-IR, homeostasis model assessment of insulin resistance; Cr, serum creatinine; eGFR, estimated glomerular filtration rate; CVD, cardiovascular disease.

Supplemental Table 5. Receiver operating characteristic analyses of TG and lipid ratios for identifying increased UACR in men and women.

| **Variables** | **AUC (95% CI)** | **Cut-off according to Youden's index** | **Sensitivity (%)** | **Specificity (%)** |
| --- | --- | --- | --- | --- |
| **Female** |  |  |  |  |
| TG | 0.588(0.578-0.597) | 1.525 | 0.522 | 0.607 |
| TG/HDL-C | 0.595(0.586-0.605) | 0.900 | 0.689 | 0.445 |
| LDL-C/HDL-C | 0.506(0.496-0.516) | 2.640 | 0.294 | 0.721 |
| Non-HDL-C/HDL-C | 0.538(0.527-0.548) | 3.376 | 0.327 | 0.731 |
| **Male** |  |  |  |  |
| TG | 0.559(0.542-0.576) | 1.785 | 0.419 | 0.677 |
| TG/HDL-C | 0.568(0.551-0.585) | 1.284 | 0.571 | 0.546 |
| LDL-C/HDL-C | 0.502(0.485-0.519) | 2.930 | 0.272 | 0.748 |
| Non-HDL-C/HDL-C | 0.527(0.510-0.544) | 3.183 | 0.505 | 0.545 |

Abbreviations: TG, triglyceride; HDL-C, high-density lipoprotein cholesterol; LDL-C, low-density lipoprotein cholesterol; non-HDL-C, non-high-density lipoprotein cholesterol.
