# Supplementary figures and images for: Triglycerides to high-density lipoprotein cholesterol ratio is superior to triglycerides and other lipid ratios as an indicator of increased urinary albumin-to-creatinine ratio in the general population of China: a cross-sectional study
Source: Lipids Health Dis. 2021 Feb 15;20:13. doi: 10.1186/s12944-021-01442-8 (PMC7883433; doi:10.1186/s12944-021-01442-8)

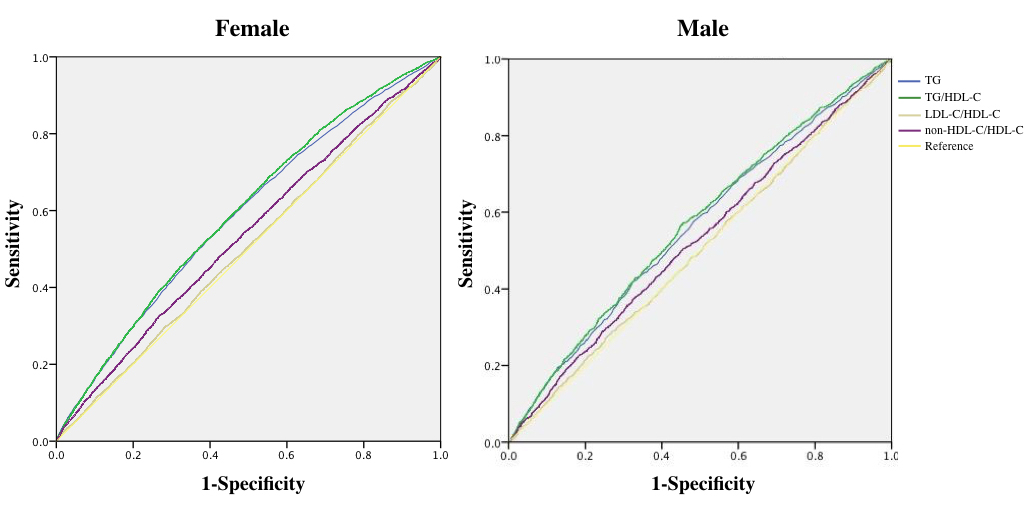

Supplement: Supplementary file 2 — Additional file 2 Figure S1. Receivers operating characteristic (ROC) curves of TG and lipid ratios for identifying increased UACR. [file 12944_2021_1442_MOESM2_ESM.jpg]
